# How Relevant Is the Place Where First-Year College Students Live in Relation to the Increase in Body Mass Index?

**DOI:** 10.3390/healthcare9121638

**Published:** 2021-11-26

**Authors:** Antonio Viñuela, Juan José Criado-Álvarez, Javier Aceituno-Gómez, Carlos Durantez-Fernández, José Luis Martín-Conty, Francisco Martín-Rodríguez, Luis Miguel Cano Martín, Clara Maestre Miquel, Begoña Polonio-López, Alicia Mohedano-Moriano

**Affiliations:** 1 Faculty of Health Sciences, University of Castilla-La Mancha, 45600 Talavera de la Reina, Spain; Javier.aceituno@uclm.es (J.A.-G.); Carlos.durantez@uclm.es (C.D.-F.); joseluis.MatinConty@uclm.es (J.L.M.-C.); clara.maestre@uclm.es (C.M.M.); begona.polonio@uclm.es (B.P.-L.); 2 Department of Medical Sciences, School of Health Sciences, University of Castilla-La Mancha, Avenida Real Fábrica de las Sedas, s/n, 45600 Talavera de la Reina, Spain; jjcriado@jccm.es (J.J.C.-Á.); alicia.mohedano@uclm.es (A.M.-M.); 3 Management of Integrated Attention of Talavera de la Reina, SESCAM, 45600 Talavera de la Reina, Spain; 4 Center for Advanced Clinical Simulation, Advanced Life Support Unit, Emergency Medical Services, Faculty of Medicine, University of Valladolid, 47005 Valladolid, Spain; fmartin@saludcastillayleon.es; 5 Menasalbas Health Center, Toledo Primary Care Management, 45128 Toledo, Spain; luismcano@msn.com

**Keywords:** adolescents, lifestyles, nutrition, overweight and obesity, food intake

## Abstract

(1) Objective: This study analyzes the evolution of the body mass index (BMI) throughout the academic year associated with changes in the lifestyle associated with the place where students live during the course, lifestyle design, and health strategies for the university community. (2) Methods: A total of 93 first-year nursing students participated in this study. Data were collected throughout the course by administering self-reported questionnaires about eating habits and lifestyles, weight, and height to calculate their BMI and place of residence throughout the course. Data were analyzed using statistical analysis (Mann–Whitney, chi-square, Student’s t-test, repeated-measures analysis of variance, and least significant difference tests). (3) Results: We found that the mean BMI increases significantly throughout the course among all students regardless of sex, age, eating habits, or where they live during the course. At the beginning of the course, the mean BMI was 22.10 ± 3.64. The mean difference between the beginning of the course and the middle has a value of *p*-value < 0.015 and between the middle of the course and the end a *p*-value < 0.009. The group that increased the most is found among students who continue to live in the family nucleus rather than those who live alone or in residence. Students significantly changed their eating and health habits, especially those who live alone or in residence. (4) Conclusions: There is an increase in BMI among students. It is necessary to carry out seminars or talks that can help students understand the importance of good eating practices and healthy habits to maintain their weight and, therefore, their health, in the short, medium, and long term and acquire a good quality of life.

## 1. Introduction

It is described in the bibliography that throughout the year there is an increase in BMI in the world population. A group of special interest are young people on their way to adulthood in which there are changes that may be more influential than others, such as changes in lifestyle and eating habits. One of these changes is the move from high school to university. Within this change, we find university students who continue to live in the same place of residence during the course, while others live in university residences or alone.

The transition from adolescence to adulthood for many young people coincides with the beginning of their first year of college. For many students, this implies the first prolonged estrangement from the family and the adoption of new responsibilities, among which one of the most important is taking care of their own nutrition [1,2]. Many students begin to live alone or share a flat, while others opt for a residency, and some continue to live in the same place as before enrolling in the university. Living alone or in a shared residence involves changes in organizational, psychological, time management, and economic behaviors, among others, many of which are new for students [3]. Those who live in a residence have fewer changes since many of their basic needs are covered, such as meals, laundry, or cleaning. Nevertheless, they do not control their own diet, as it is imposed by the residence. On the other hand, those who continue to live in the family home experience fewer changes, mainly those related to the new place of study and the responsibility of starting university. Several authors have described how attachment to family and friends decreases among students who decide to live alone, being replaced by new friends, and adapting to a new environment [4,5].

Several factors are involved in the increase in BMI (a simple, inexpensive, non-invasive and rapid measure [6]) among adolescents and young adults, with a greater tendency to gain lean mass in boys compared with girls [7]. Living in the same house as in previous stages of life, living in a university residence, or living alone or sharing a flat may have an impact on the weight gain of students. However, other factors may play a role, such as changes in eating habits, excessive calorie intake, a sedentary lifestyle, or irregular mealtimes [8,9]. Little is known about whether the type of residence significantly affects this increase in BMI.

Food quality, changes in the number of meals, lower intake of fruits and vegetables, and increased consumption of fast food, along with lifestyle changes, can lead to increased BMI, which leads to the higher probability of suffering from health problems such as obesity or cardiovascular diseases, among others [2,10,11,12,13].

In the transition from high school to college, students acquire less healthy nutritional habits [14,15]. The first year is characterized by a decrease in the consumption of fruits/vegetables and an increase in the intake of foods rich in sugar, sodium, and saturated fat [16]. A balanced diet has been shown to contribute to stable health and mental state, and thus better academic performance [17,18]. These imbalances are accompanied by inadequate food intake and little or no physical activity, along with poor sleep habits and nutrient-poor diets [19]. Increased consumption of tobacco and alcohol has also been shown, which would also suggest a decrease in their quality of life [14,18,20].

The main objective of the study is to find out how the changes in the life of students according to the place of residence influence the first year and analyze the increase in changes in BMI. For this, we evaluated the students in three groups, according to the place where they lived during the course, as well as their self-perceived health and the consumption of tobacco–alcohol, being factors that have been identified with an increase in BMI [21,22,23]. We will also analyze the eating patterns and habits of students, which are known to many to pose numerous challenges during the first year of university [24].

## 2. Materials and Methods

### 2.1. Study Design and Participants

This is a longitudinal study carried out on newly enrolled nursing students at the Faculty of Health Sciences of Talavera de la Reina, one of the campuses of the University of Castilla-La Mancha (UCLM). All new students were included in the study. The only exclusion criterion was the refusal to participate by any of the students. No other exclusion criteria were established for the very objective of the project. They were informed about the project orally and in writing. All participants were given the documentation on the study, informed of the voluntary nature of their participation, and asked to sign an informed consent form. Of the 100 students enrolled, 93 agreed to participate. The sample consisted of 80 women (86.0%) with a mean age of 20.23 ± 4.26 and 13 men (14.0%) with a mean age of 18.46 ± 0.88. The global mean age was 19.98 ± 4.01 years.

### 2.2. Data Collection and Schedule

The study design was longitudinal and based on where new students lived during their first academic year and their BMI variation. They were divided into three groups: those who lived in the same house as before starting university, those who lived in a residence hall, and those who lived in a flat alone or shared with others.

Three “ad hoc” questionnaires designed to obtain data on lifestyle and habits were administered. The second questionnaire was administered in February at the end of the first semester and the last one immediately before the end of the course prior to the exams. The data collected included information on sex, age, and anthropometric data, and place of residence.

The students’ BMI (kg/m^2^) was calculated using self-reported height and weight. Self-reported BMI has been used extensively in previous studies. BMI was measured according to the BMI criteria established by the World Health Organization (WHO) (World Health Organization, 1998).

### 2.3. Data on Healthy Habits

The surveys collected data on tobacco and alcohol consumption and frequency of use.

They also asked about physical activity performed in gyms or outdoors.

### 2.4. Statistical Analysis

Data analysis was performed using SPSS for Windows (Statistical Package Social Sciences version 15.0). For the statistical analysis, the parameters were used according to the measurement scales of the variables (simple frequencies, measures of central tendency, and standard deviations).

We used the Student’s t-test when the dependent variable was continuous with a normal distribution, e.g., BMI, versus a dichotomous independent variable such as sex. We used the chi-square test when the two variables were dichotomous such as sex and smoking or were nominal.

Statistical tests were used depending on the type of dependent and independent variables, and whether they were normally distributed (parametric statistics) or non-normally distributed (non-parametric statistics). The parameters were used according to the measurement scales of the variables (simple frequencies, measures of central tendency, and standard deviations). For example, for the statistical analysis of the independent variables, an ANOVA was performed for the relationship between the continuous normal variable and the nominal variable. In the cases of dichotomous variables, we use the Student’s t-test. A chi-square test was used to compare the nominal and dichotomous variables.

The Kolmogorov–Smirnov test was used to study the normality distribution of the variables, to show that the distributions did not deviate significantly from normal. For the statistical analysis of the independent variables, an ANOVA was performed for the relationship between the continuous normal variable and the nominal variable. In the cases of dichotomous variables, we used the Student’s t-test. A chi-square test was used to compare the nominal and dichotomous variables. The confidence interval was set at 95.0%.

## 3. Results

### 3.1. Sample Characteristics

This longitudinal study was conducted with a sample of 93 respondents, all of whom were first-year undergraduate nursing students. The sample consisted of 80 women (86.0%), with a mean age of 20.23 ± 4.26 years, and 13 men (13.0%), with a mean age of 18.46 ± 0.88 years. The mean age was slightly higher in girls, the global mean age being 19.99 ± 3.99 years. Table 1 shows the distribution, percentages, and standard deviations of the places where the students lived during the year: 21 students (22.6%) continued to live in the same house as before starting university, 23 (24.7%) lived in residences, and 49 (52.7%) lived alone or in shared flats. These numbers did not vary during the academic year. A little more than half of the students chose to live alone or share a flat. The table also shows the means and standard deviations of the students’ BMI scores for the year. At the beginning of the academic year, the mean BMI was 22.10 ± 3.64. The mean of the independent samples of the changes in BMI varied, with the change from BMI-1 to BMI-2 (*p*-value < 0.015) and between BMI-2 and BMI-3 (*p*-value < 0.009) being statistically significant. By gender, men had a mean BMI of 23.33 ± 5.07 and the mean BMI of women was slightly lower than 21.90 ± 3.38. These data are not statistically significant (*p* > 0.05), *p* = 0.251. The mean BMI between men and women increased during the academic year in the three measurement moments, being greater between the first and second measurement than between the second and third. These data are observed in Table 2 and Table 3.

It is worth noting that the students’ BMIs spanned all ranges. A total of 12 of the students presented values that represented low weight (16.4% of the total). However, most of the students corresponded to what is known as normal weight, 45 students (61.6%), while 17.8% of the sample were overweight and obese, a total of 3 students (4.2%). At the end of the course, these values varied, observing that in all cases the weights shifted towards weight gain, which is why we find the total number of students in healthy weight is 55 (61.1%), overweight is 18 (20.0%), and obesity is 8 students (8.9%). The only case in which we see a decrease in students is underweight, 9 (10.0%) cases. The data of the subjects can be observed in Table 4 where they can be seen as they vary depending on the values of the body mass index.

The Student’s *t*-test used to analyze the differences in means between men and women revealed a significant difference between men and women in the variation between BMI II and III scores. No significant differences related to gender were found for the other variables.

The ANOVA performed to examine the relationship between the type of residence and the variation in weight showed that the place where the students lived had no impact on the variation in BMI between the first and last rounds of the survey.

Therefore, we did not find statistically significant evidence that the type of residence is a cause of an increase in BMI in students throughout the academic year. However, the chi-square test on BMI and type of residence did reveal a statistically significant association between students with a higher BMI, those whose values represented type I and II obesity, and who lived in the family home, *p* < 0.05 (*p* = 0.49).

Furthermore, we did not find gender-related differences between the increase in BMI and the place of residence. BMI increased similarly in both groups of students, regardless of gender, *p* > 0.05.

### 3.2. Health Condition

Students were asked about their perception of their health status at the beginning and end of the academic year. At first, 22 first-year students (23.7%) perceived their health as very good, 40 (43.0%) reported that their health was good, 8 (8.6%) said that their health was average, and the 23 remaining students (24.7%) either did not answer the question or could not assess their health status. Once the academic year ended, the students were asked about their health again and 44 students (47.3%) reported that their health status was the same as at the beginning of the course, 23 (24.7%) that their health status had improved, and 17 (18.3%) perceived their health to be worse. The remaining 7 students (9.7%) did not answer the question or could not assess their health status. It should be noted that almost half of the students reported feeling that their health was the same as at the beginning of the year, which implies that their lifestyle had remained constant throughout the academic year. However, 13 students (14.0%) were smokers or started smoking during the year and 14 (15.1%) reported drinking more alcohol than at the beginning of the academic year. In Table 5, we show the perception of the health status of the students at the beginning and at the end of the study. Its evolution can be observed.

We found no association in the self-perceived health of the students during the academic year. Most students (48.5%) perceived that their health was the same throughout the year, compared with 23.5% who reported feeling better, 23.5% who perceived their health status as worse, and 4.5% who did not respond. Pearson’s chi-square analysis was not statistically significant *p* > 0.05 (*p* = 0.713), rejecting the relationship proposed between the type of residence and the increase in BMI. Data not shown.

### 3.3. Nutritional Condition

The data shown below are reflected in Table 5. The participants were asked if they had made changes in terms of nutrition during the year, to which 74 (79.6%) answered that their nutritional habits had not changed. A total of 17 (18.3%) reported having started a diet or simply having incorporated more fruits or vegetables into their diet. Of these students, 15 (88.2%) were women and only two were men. Two of the participants did not answer the question.

Regarding whether they engaged in physical activity, 45 students (48.4%) reported going to the gym regularly during the academic year, while the remaining 46 (49.5%) did not go to the gym at all.

Regarding alcoholic beverages, 14 students (15.1%) said that they drank alcohol frequently while 77 (82.8%) did not report alcohol consumption.

## 4. Discussion

The students’ BMI increased from start to finish in their first year of study. This is consistent with studies in other student populations, where BMI values in the population have been shown to increase year after year, regardless of age and social or economic status [7,8,25,26,27]. College students adopt new lifestyles that involve higher caloric intake and a sedentary lifestyle, causing an increase in body weight. The increase in BMI is most evident towards the end of the academic year, which could be related to a more sedentary lifestyle and the pressure of final exams.

A healthy diet has been shown to play a key role in the lives of college students [28]. Those who live at home with their parents may find that their diet is more controlled. The group of students who live alone is possibly more affected by their type of residence and could be more prone to a greater increase in BMI. However, the individual analysis of each group did not reveal significant findings except in those who continue to live with their parents, which leads us to determine that other factors are the cause of this increase in BMI.

Few studies have examined the impact of the emergence of new responsibilities such as socioeconomic status [29,30]. Our data reveal a greater increase in BMI in boys than in girls, which is consistent with studies conducted in different populations and ages [31,32]. Changes in lifestyle associated with reaching the age of majority, lower levels of parental control, and the independence of living alone can generate these changes in eating habits that, in turn, can lead to weight gain in short periods, such as an academic year. However, a statistically significant increase in BMI was detected in students who did not change their place of residence in the transition from high school to university, compared with those who live alone or in residences. Students residing in the dormitories have their dietary needs guaranteed even though they may skip meals. For the last group, those who live alone, despite eating and making their own food, the quality of the meals or the repetition of types of food based on pre-cooked dishes or poor-quality foods can be the cause of weight gain. However, a reasonable number of our participants in all groups reported having acquired better eating habits, with higher consumption of fruits and vegetables.

An increase in alcohol and tobacco consumption was observed in all groups. Some studies have shown an association between smoking, low levels of physical activity, and an increase in obesity [33,34] while other studies have found a relationship between obesity and alcohol intake regardless of sex and have shown that the BMI is inversely related to physical activity in women and to self-perceived health in men [34]. On the contrary, several studies have not found an association between lack of physical activity, a sedentary lifestyle, alcohol and tobacco consumption, and weight gain [21,27]. According to these authors, first-year college students are young people who are still growing, which would explain the lack of direct association between these determinants and weight gain. Our data, however, lead us to believe that lower levels of activity and increased sedentary behaviors, along with inappropriate eating habits, are factors that influence weight gain, at least among our students. The results presented in this work suggest a slight weight gain among students who continue to live in the family home, regardless of age and sex. This trend may be a consequence of their food supply being catered and, therefore, they show less concern about what they eat, compared with those who oversee managing both the kitchen and their finances. It is possible that a life with basic needs covered (food, cleaning, and expenses) causes students to have a lower rate of stress and have a lower metabolic rate by increasing their BMI. These data must be contrasted in future studies.

It would be necessary to continue observing these students throughout the following academic years and examine how their weight and physical activity evolved and the changes in their eating and health habits. Authors should discuss the results and how they can be interpreted from the perspective of previous studies and of the working hypotheses. The findings and their implications should be discussed in the broadest context possible. Future research directions may also be highlighted.

A limitation of the study is the self-report. Some bias related to self-reported information has been shown to exist, but the data are considered adequate for research purposes Estimated BMI values may result in lower measurements than those based on directly measured weight and height [21,28]. Other authors have found a high level of precision in data from self-reported surveys and direct measurements. The data are reliable since we administered three surveys at three points during the year, where there was no variation in the height values, but the weight values did change. The respondents had no reason to make mistakes in their height and weight measurements.

The longitudinal design of our study meant that we lost some of the students during the year. The design is also a strength as it allowed us to see the evolution of BMI in a sufficient number of students to obtain meaningful data on the cause/effect of the increase in BMI. In addition, we received data from 93.0% of the survey participants.

First-year nursing students study nutrition, which could have led them to change their dietary behavior, improve their consumption of fruits and vegetables, reduce their intake of sugary drinks, and increase physical activity. Such changes were observed in the students towards the end of the academic year.

## 5. Conclusions

There is a statistically significant increase in BMI among students who continue to live with their relatives. This is probably because they have their basic needs covered, including food, finances, cleaning, etc. They are not generating stress for these reasons while their other colleagues must solve these issues together with the completion of the assessment tests.

The study revealed an increase in BMI in our study sample among first-year students. It occurs among all students regardless of age and sex (note: we removed socioeconomic status). Among the variables that explain this increase, it seems that those who continue to live in the same family nucleus have a greater increase than the rest who live alone or in residences.

It is necessary to delve deeper into the habits of these groups of students to determine the cause of this increase in BMI. Additional studies with a larger number of students are needed to confirm these data in successive studies.

## Figures and Tables

**Table 1:** Characteristics of student population in the study sample.

Variable	Total	Girls	Boys
N (%)	93 (100%)	80 (86.0%)	13 (14.0%)
Age (years)	19.99 ± 3.99	20.23 ± 4,26	18.46 ± 0.88
Residence along the course	Living with their parents	Living in residence	Living alone or sharing a flat
N	21	23	49
%	22.6%	24.7%	52.7%
BMI			
BMI-1 beginning course	Girls	Boys	*p*-value
	21.98 ± 3.67	23.88 ± 5.19	*p* < 0.156
BMI-2 mid-course	Girls	Boys	
	22.39 ± 4.09	25.61 ± 5.69	*p* < 0.015
BMI-3 at the end of the course	Girls	Boys	
	22.64 ± 4.23	26.28 ± 6.05	*p* < 0.009
BMI: Body mass index			

**Table 2:** BMI and place where students lived during the course; Descriptive statistics; Kolmogorov–Smirnov test by sample.

Variable			N	Mean	SD	*p*-Value
BMI-1	living alone		39	21.94	±2.83	0.733
beginning course	living at residence		21	22.78	±4.27	
	living at home with their parents		14	22.27	±5.83	
	Total		74	22.24	±3.92	
BMI-2	living alone		49	22.49	±3.16	0.660
mid-course	living at residence		22	23.00	±4.92	
	living at home with their parents		20	23.55	±6.44	
	Total		91	22.85	±4.46	
BMI-3	living alone		48	22.83	±3.35	0.612
At the end of the course	living at residence		22	23.05	±4.84	
	living at home with their parents		21	24.04	±6.80	
	Total		91	23.16	±4.67	
	**N**	**Media**	**Standard Deviation**		**Minimum**	**Maximum**
Age	93	19.98	4.01		17	41
BMI-1	74	22.24	3.92		13.88	35.90
BMI-2	91	22.85	4.46		16.10	37.55
BMI-3	91	23.16	4.67		15.45	39.86
			**Age**	**BMI-1**	**BMI-2**	**BMI-3**
N		93		74	91	91
normalis parametri ^a,b^		media	19.98	22.24	22.85	23.16
		Standard deviation	4.01	3.92	4.46	4.67
plures extremae differentiae		absolute	0.29	0.01	0.11	0.10
		positivum	0.27	0.01	0.11	0.10
		negans	−0.29	−0.08	−0.08	−0.07
Kolmogorow–Smirnov Z			2.79	0.85	1.02	0.97
Asintot. Sig. (bilateral)			0.00	0.46	0.25	0.30

S.D. = standard deviation; N = number of subjects; a = the contrast distribution is normal; and b = they have been calculated from the data. BMI =body mass index.

**Table 3:** The ANOVA test with repeated measures was used, for the three measures of BMI and for the sex; Descriptive statistics; Mauchly sphericity test; estimated marginal means; Peer comparison.

Variable	Sex	Media			Standard Deviation		N
IMC-1	Boys	23.88			5.189		10
	Girls	22.19			3.68		60
	Total	22.43			3.93		70
IMC-2	Boys	25.07			5.51		38
	Girls	22.36			3.93		10
	Total	22.74			4.25		70
IMC-3	Boys	25.26			4.94		10
	Girls	22.63			4.03		60
	Total	23.0083			4.24		70
**Intra-Subject Effect**	**W De Mauchly**	**Chi-Square Approx**	**Gl**	**Signification**	**Epsilon(a)**		
	Greenhouse–Geisser	Huynh–Feldt	Lower limit	Greenhouse–Geisser	Greenhouse–Geisser	Huynh–Feldt	Lower limit
factor1	0.910	6.30	2	0.043	0.918	0.956	0.500
			**Confidence Interval for the 95% Mean**				
Sex	Media	Typical error	Lower limit			Upper limit	
Boy	24.74	1.27	22.20			27.27	
Girl	22.39	0.52	21.36			23.43	
					**Confidence Interval for the 95% Mean**		
(I) Sex (J) sex	Difference between Medias (I-J)	Typical error	Significance ^a^		Lower limit Upper limit		
Boy–Girl	2.34	1.37	0.09		−0.39	5.08	
Girl–Boy	−2.34	1.37	0.09		−0.508	0.39	

The table contrasts the null hypothesis that the error covariance matrix of the transformed dependent variables is proportional to an identity matrix. a = used to correct degrees of freedom in averaged significance tests. The corrected evidence is shown in the table as evidence for inter-subject effects. = design: intersection + where you live. Intra-subject design: factor1. Based on estimated marginal means. A =adjustment for multiple comparisons: Bonferroni.

**Table 4:** **Data** of subjects as a function of body mass index values.

BMI	N	%	N	%
	Beginning of course		The end of course	
Underweight	12	16.4%	9	10.0%
Healthy weight	45	61.6%	55	61.1%
Overweight	13	17.8%	18	20.0%
Obesity	3	4.3%	8	8.9%

N = number of subjects.

**Table 5:** Indicator of perception of their state of health at the beginning and end of course.

**Beginning of Course** **N, (%)**	Heath to be very good	Health as good	Health was average	Do not know, no answer	
	22 (23.7%)	40 (43.0%)	8 (8.6%)	23 (24.7%)	
**The end of Course** **N, (%)**	Reporting their health was the same as at the start of the year	Perceived their health to be the same throughout the year	Their state of health had improved	Perceiving their health as worse	Do not know, any answer
	44 (47.3%)	45 (48.5%)	23 (24.7%)	17 (18.3%)	7 (9.7%)
**Other Changes Throughout the Year** **N, (%)**	Smokers or began to smoke	Drinking more alcohol than at the start of course	Changes in nutrition	Reported going to the gym regularly	
	13 (14.0%)	14 (15.1%)	74 (79.6%)	45 (48.4%)	

## Data Availability

The study did not report any data.

## References

[1] Chourdakis M., Tzellos T., Papazisis G., Toulis K., Kouvelas D. (2010). Eating habits, health attitudes and obesity indices among medical students in northern Greece. Appetite.

[2] Hilger J., Loerbroks A., Diehl K. (2017). Eating behaviour of university students in Germany: Dietary intake, barriers to healthy eating and changes in eating behaviour since the time of matriculation. Appetite.

[3] Morseth B., Jørgensen L., Emaus N., Jacobsen B.K., Wilsgaard T. (2011). Tracking of Leisure Time Physical Activity during 28 yr in Adults. Med. Sci. Sports Exerc..

[4] Davis J. (2013). Crossing Customs: International Students Write on US College Life and Culture.

[5] Dyson R., Renk K. (2006). Freshmen adaptation to university life: Depressive symptoms, stress, and coping. J. Clin. Psychol..

[6] Rogol A.D., Clark P.A., Roemmich J.N. (2000). Growth and pubertal development in children and adolescents: Effects of diet and physical activity. Am. J. Clin. Nutr..

[7] Himes J.H., Dietz W.H. (1994). Guidelines for overweight in adolescent preventive services: Recommendations from an expert committee. Am. J. Clin. Nutr..

[8] Vadeboncoeur C., Townsend N., Foster C. (2015). A meta-analysis of weight gain in first year university students: Is freshman 15 a myth?. BMC Obes..

[9] Abarca-Gómez L., Abdeen Z.A., Hamid Z.A., Abu-Rmeileh N.M., Acosta-Cazares B., Acuin C., Adams R.J., Aekplakorn W., Afsana K., Aguilar-Salinas C.A. (2017). Worldwide trends in body-mass index, underweight, overweight, and obesity from 1975 to 2016: A pooled analysis of 2416 population-based measurement studies in 128.9 million children, adolescents, and adults. Lancet.

[10] Musaiger A.O., Bader Z., Al-Roomi K., D’Souza R. (2011). Dietary and lifestyle habits amongst adolescents in Bahrain. Food Nutr. Res..

[11] El-Gilany A.-H., Elkhawaga G. (2012). Socioeconomic determinants of eating pattern of adolescent students in Mansoura, Egypt. Pan Afr. Med. J..

[12] Mousa T.Y., Al-Domi H.A., Mashal R.H., Jibril M.A.-K. (2010). Eating disturbances among adolescent schoolgirls in Jordan. Appetite.

[13] Deliens T., Deforche B., De Bourdeaudhuij I., Clarys P. (2015). Determinants of physical activity and sedentary behaviour in university students: A qualitative study using focus group discussions. BMC Public Health.

[14] Cano Martín L.M., Gonzalez-Gonzalez J., Mohedano-Moriano A., Viñuela ACriado-Álvarez J.J. (2019). Estado nutricional de una población escolar en España y su relación con hábitos de alimentación y actividad física. Arch Latinoam Nutr..

[15] Ludy M.-J., Tan S.-Y., Leone R.J., Morgan A.L., Tucker R.M. (2018). Weight gain in first-semester university students: Positive sleep and diet practices associated with protective effects. Physiol. Behav..

[16] Breslow R.A., Guenther P., Smothers B.A. (2006). Alcohol Drinking Patterns and Diet Quality: The 1999–2000 National Health and Nutrition Examination Survey. Am. J. Epidemiol..

[17] Gorgulho B., Marchioni D.M.L., da Conceição A.B., Steluti J., Mussi M.H., Nagai-Manelli R., Teixeira L.R., da Luz A.A., Fischer F.M. (2012). Quality of diet of working college students. Work.

[18] St-Onge M.-P., Roberts A.L., Chen J., Kelleman M., O’Keeffe M., Roychoudhury A., Jones P. (2011). Short sleep duration increases energy intakes but does not change energy expenditure in normal-weight individuals. Am. J. Clin. Nutr..

[19] Deliens T., Clarys P., De Bourdeaudhuij I., Deforche B. (2014). Determinants of eating behaviour in university students: A qualitative study using focus group discussions. BMC Public Health.

[20] Al-Hazzaa H.M., Musaiger A.O., Abahussain N.A., Al-Sobayel H.I., Qahwaji D.M. (2013). Lifestyle correlates of self-reported sleep duration among Saudi adolescents: A multicentre school-based cross-sectional study. Child Care Health Dev..

[21] Henderson R.M. (2005). The bigger the healthier: Are the limits of BMI risk changing over time?. Econ. Hum. Biol..

[22] Linares C., Su D. (2005). Body mass index and health among Union Army veterans: 1891–1905. Econ. Hum. Biol..

[23] Banna J.C., Buchthal O.V., Delormier T., Creed-Kanashiro H.M., Penny M.E. (2015). Influences on eating: A qualitative study of adolescents in a periurban area in Lima, Peru. BMC Public Health.

[24] Al-Kilani H., Waly M., Yousef R. (2012). Trends of obesity and overweight among college students in Oman: A cross sectional study. Sultan Qaboos Univ. Med J..

[25] Nojomi M., Najamabadi S. (2006). Obesity among university students, Tehran, Iran. Asia Pac. J. Clin. Nutr..

[26] Trujillo-Hernández B., Vásquez C., Almanza-Silva J.R., Jaramillo-Virgen M.E., Mellin-Landa T.E., Valle-Figueroa O.B., Pérez-Ayala R., Millán-Guerrero R.O., Prieto-Díaz-Chávez E., Newton-Sánchez O. (2010). Frecuencia y factores de riesgo asociados a sobrepeso y obesidad en universitarios de Colima, México. Revista Salud Públ..

[27] Nola I.A., Jelinić J.D., Matanić D., Pucarin-Cvetković J., Marković B.B., Senta A. (2010). Differences in eating and lifestyle habits between first- and sixth-year medical students from Zagreb. Coll. Antropol..

[28] Chao C.-Y., Shih C.-C., Wang C.-J., Wu J.-S., Lu F.-H., Chang C.-J., Yang Y.-C. (2014). Low socioeconomic status may increase the risk of central obesity in incoming university students in Taiwan. Obes. Res. Clin. Pr..

[29] Seubsman S.-A., Lim L.L.-Y., Banwell C., Sripaiboonkit N., Kelly M., Bain C., Sleigh A. (2010). Socioeconomic Status, Sex, and Obesity in a Large National Cohort of 15–87-Year-Old Open University Students in Thailand. J. Epidemiol..

[30] Mayer M., Gleiss A., Haeusler G., Borkenstein M., Kapelari K., Köstl G., Lassi M., Schemper M., Schmitt K., Blümel P. (2014). Weight and body mass index (BMI): Current data for Austrian boys and girls aged 4 to under 19 years. Ann. Hum. Biol..

[31] Bener A., Kamal A.A. (2005). Growth patterns of Qatari school children and adolescents aged 6–18 years. J. Health Popul. Nutr..

[32] Di Milia L., Vandelanotte C., Duncan M.J. (2013). The association between short sleep and obesity after controlling for demographic, lifestyle, work and health related factors. Sleep Med..

[33] Lahti-Koski M., Pietinen P., Heliövaara M., Vartiainen E. (2002). Associations of body mass index and obesity with physical activity, food choices, alcohol intake, and smoking in the 1982–1997 FINRISK Studies. Am. J. Clin. Nutr..

[34] Brener N.D., Eaton D.K., Lowry R., McManus T. (2004). The Association between Weight Perception and BMI among High School Students. Obes. Res..

